# 血清细胞角蛋白19片段与胸腺肿瘤临床病理特征和预后的相关性研究

**DOI:** 10.3779/j.issn.1009-3419.2018.07.03

**Published:** 2018-07-20

**Authors:** 雪飞 章, 春宇 吉, 志涛 谷, 文涛 方

**Affiliations:** 200030 上海，上海交通大学附属胸科医院 Department of Thoracic Surgery, Shanghai Chest Hospital, Shanghai Jiaotong University, Shanghai 200030, China

**Keywords:** 胸腺肿瘤, 细胞角蛋白19片段, 肿瘤分期, 组织学类型, 预后, Thymic epithelial tumors, Cyfra 21-1, Tumor stage, Histotype, Prognosis

## Abstract

**背景与目的:**

胸腺肿瘤中尚无肿瘤标志物的应用，本研究旨在评估血清细胞角蛋白19片段（cytokeratin 19 fragment, Cyfra 21-1）与胸腺肿瘤临床病理特征和预后的相关性。

**方法:**

回顾性分析上海市胸科医院2012年11月-2016年9月收治的159例胸腺肿瘤病例的临床资料。比较术前血清Cyfra 21-1水平在不同分期及组织学类型间的差异，研究术前及术后血清Cyfra 21-1水平与复发的关系。

**结果:**

在局部晚期（T4期）（*P* < 0.001）、胸腺癌（*P* < 0.001）患者中，术前血清Cyfra 21-1水平显著升高，且当术前Cyfra 21-1≥1.66 ng/mL时，其对术后复发有提示意义。分析患者随访的血清Cyfra 21-1，将2.66 ng/mL作为cut-off值时，提示胸腺肿瘤复发的敏感度为0.667，特异度为0.925，阳性预测值为0.462，阴性预测值为0.966。

**结论:**

术前患者血清Cyfra 21-1水平较高，有助于提示肿瘤分期较晚、肿瘤恶性程度较高，或可提示术后复发的风险升高。在随访时结合血清Cyfra 21-1检测，将可能有助于提高复发患者的检出率，改善预后。

## Abstract (en)

**Background and objective:**

So far there's no tumor maker applied in diagnosis and treatment of thymic epithelial tumors. This study is to assess the correlation between serum cytokine 19 fragment (Cyfra 21-1) and clinicopathological features and prognosis of thymic epithelial tumors (TETs).

**Methods:**

The clinical data of 159 patients with TETs in Shanghai Chest Hospital was retrospectively analysed. Patients were divided into groups according to different tumor stages and histotypes. Serum Cyfra 21-1 was thus compared. In addition, the possible relationship between perioperative serum Cyfra 21-1 level and the recurrent status was carrid out.

**Results:**

Preoperative Cyfra 21-1 serum concentrations in patiants with advanced stage (T4) and thymic carcinomas were significantly higher than that in others (*P* < 0.001, *P* < 0.001, respectively). When the preoperative serum level exceeds the out-off of 1.66 ng/mL, it possibly indicates the recurrence during follow up. Furthermore, the sensitivity, specificity, and positive as well as negative predictive value (PPV and NPV) of postoperative Cyfra 21-1 to predict tumor recurrence were evaluated. At a cut-off of Cyfra 21-1 of 2.66 ng/mL, the sensitivity was 0.7, the specificity was 0.925, the PPV was 0.5 and the NPV was 0.966.

**Conclusion:**

The elevated level of preoperative serum Cyfra 21-1 indicates an advanced stage of tumor or a more malignant histotype (thymic carcinoma). It also probably suggests a higher risk of tumor recurrence. During the oncological follow up, in addition to regular imaging examinations, the blood test of serum Cyfra 21-1 is also suggested to improve the diagnosis of tumor recurrence in order to improve the prognosis.

胸腺肿瘤（thymic epithelial tumors, TETs）是一种起源于胸腺上皮的实体性肿瘤，不包括起源于生殖细胞、淋巴细胞、神经内分泌细胞及脂肪组织的肿瘤，在胸部肿瘤中相对罕见，约占前纵隔肿瘤的50%。按照上皮细胞形态及淋巴细胞和上皮细胞比例，2004版世界卫生组织（World Health Organization, WHO）组织学分类将其分为胸腺瘤（A型、AB型、B1型、B2型、B3型及少量其他类型）（thymoma）和胸腺癌（thymic carcinoma, Tca）。国际上报道的发病率为1.3/100万人-3.2/100万人。美国医疗保险监督、流行病学和最终结果（Surveillance, Epidemiology, and End Results, SEER）数据库中显示，胸腺肿瘤在亚裔中发病率（2.5/100万）高于白人（1.0/100万）^[1, 2]^。因此国内的人群发病率可能略高于上述比例。目前为止，手术切除仍是胸腺肿瘤治疗的主要方法^[3, 4]^，而治疗前肿瘤分期、WHO组织学分型及手术切除状态是明确的胸腺肿瘤预后的影响因素^[5, 6]^。另一方面，肿瘤标志物逐渐成为肿瘤早期诊断、复发监测、疗效及预后判断的重要参考指标。其测定具有简便、微创、价廉等优点，已成为肿瘤研究领域的重点之一。但目前胸腺肿瘤并无特异性的肿瘤标志物。基于此，本研究回顾性分析了159例胸腺肿瘤患者血清细胞角蛋白19片段（cytokerantin-19-fragment, Cyfra 21-1）在不同肿瘤分期、组织学类型及复发状态间的差异，旨在为胸腺肿瘤患者术前评估及术后监测提供一定参考。

## 1 材料与方法

### 1.1 材料

本研究纳入了上海交通大学附属上海市胸科医院2012年11月1日-2016年9月30日直接手术的胸腺肿瘤病例159例，病理结果均由手术标本石蜡切片证实。病理分型按2004版WHO组织学分类，病理分期按美国癌症联合委员会（American Joint Committee on Cancer, AJCC）/国际癌症联盟（Union for International Cancer Control, UICC）第8版肿瘤-淋巴结-转移（tumor-node-metastasis, TNM）分期。

### 1.2 方法

资料收集：临床资料：年龄、性别；实验室资料：Cyfra 21-1采用流式荧光法检测，检测仪器为液态悬浮芯片系统Luminex 200，临床 > 5 ng/mL视为阳性。病理学资料：肿瘤组织学类型、肿瘤TNM分期、肿瘤长径。复发按国际胸腺肿瘤协作组织（International Thymic Malignancies Interest Group, ITMIG）推荐定义^[7]^，术后随访资料完整的102例患者中，共9例复发，7例依据随访胸部计算机断层扫描（computed tomography, CT）或正电子发射型计算机断层显像（positron emission computed tomography, PET）/CT诊断，2例有复发病理确诊。

### 1.3 统计学方法

数据分析采用SPSS 20.0软件，计量资料由均数±标准差（Mean±SD）表示，采用*t*检验或单因素*ANOVA*分析；分类资料采用卡方检验；对无复发生存（freedom from recurrence, FFR）采用*Kaplan-Meier*及*Cox*回归分析。双侧*P* < 0.05为差异有统计学意义；图表制作采用graphpad prism 6.0软件。

## 2 结果

### 2.1 血清Cyfra 21-1与组织学类型

病例资料及不同组间血清Cyfra 21-1水平差异如表 1所示。术前血清Cyfra 21-1水平在胸腺癌中显著高于胸腺瘤[(3.93±3.84) *vs* (1.55±1.84), *P* < 0.001]，胸腺瘤中各亚型间无统计学差异（*P*=0.364）（图 1A）。

**1:** 不同临床病理分组中血清Cyfra 21-1水平的差异 Patient characteristics according to Cyfra 21-1 serum concentrations

Characteristic	*n*	Cyfra 21-1 (ng/mL, Mean±SD)	*P*
Age (yr)			0.165
< 53	78	1.67±2.54	
≥53	81	2.22±2.36	
Gender			0.213
Male	92	2.16±3.03	
Female	67	1.67±1.30	
Histotype			0.000
Thymoma	132	1.55±1.84	
Tca^*^	27	3.93±3.84	
T stage			0.000
T1-T3	151	1.67±2.14	
T4	8	6.23±5.18	
N stage			0.004
N0	151	1.89±2.30	
N1-N2	8	4.51±6.16	
M stage			0.306
M0	152	1.99±2.51	
M1a	7	2.99±5.31	
Tumor size (mm)			0.091
> 50	87	2.25±3.06	
≤50	72	1.59±1.36	
Resection status			0.108
R0	147	1.86±2.33	
R1-R2	12	3.05±3.65	
Tca: thymic carcinoma.			

**1:**
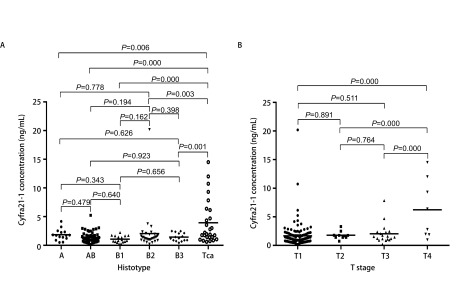
不同组织学类型及T分期中血清Cyfra 21-1水平的差异。A：胸腺瘤各亚型间血清Cyfra 21-1水平无显著差异（*P* > 0.05），而胸腺癌血清Cyfra 21-1水平显著升高（*P* < 0.05）；B：T1-T3期血清Cyfra 21-1水平无显著差异（*P* > 0.05），而T4期血清Cyfra 21-1水平显著升高（*P* < 0.001）。 Different serum Cyfra 21-1 levels in different histotypes and T stages. A: serum Cyfra 21-1 levels in subtypes of thymoma were not significantly different from each other (*P* > 0.05), while the serum Cyfra 21-1 level in thymic carcinoma was significantly higher (*P* < 0.05); B: serum Cyfra 21-1 levels in stage T1 to T3 were not significantly different from each other (*P* > 0.05), while the serum Cyfra 21-1 level in stage T4 was significantly higher (*P* < 0.001).

### 2.2 血清Cyfra 21-1与肿瘤分期

按照AJCC/UICC第8版TNM分期，术前血清Cyfra21-1水平在T4期患者高于T1-T3期患者[(6.23±5.18) *vs* (1.67±2.14), *P*=0.000]，在有淋巴结转移（N1-N2）患者高于无淋巴结转移（N0）患者[(4.51±6.16) *vs* (1.89±2.30), *P*=0.004]，而与是否存在胸膜转移无关[(2.99±5.31) *vs* (1.99±2.51), *P*=0.306]（图 1B）。

### 2.3 术前血清Cyfra 21-1关于复发状态的受试者工作特征曲线（receiver operating characteristic curve，ROC曲线）

作关于术前血清Cyfra 21-1的ROC曲线，得到曲线下面积（area under curve, AUC）为0.778±0.078，*P* < 0.05（图 2A）。取Cyfra 21-1值为1.66 ng/mL时，约登指数最大。此时敏感度为0.889（8/9），特异度为0.677（63/93），阳性预测值（positive predictive value, PPV）为0.211（8/38），阴性预测值（negative predictive value, NPV）为0.984（63/64）。据此值将患者分为高Cyfra 21-1组（血清Cyfra 21-1≥1.66 ng/mL）和低Cyfra 21-1组（血清Cyfra 21-1 < 1.66 ng/mL）。

**2:**
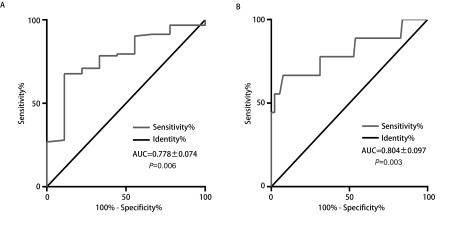
术前及术后血清Cyfra 21-1的ROC曲线。A：术前血清Cyfra 21-1的ROC曲线。曲线下面积为0.778±0.074（*P*=0.006）。当取1.66 ng/mL为cutoff值时，敏感性为0.889，特异性为0.677；B：术后血清Cyfra 21-1的ROC曲线。曲线下面积为0.804±0.097（*P*=0.003）。当取2.66 ng/mL为cutoff值时，敏感性为0.667，特异性为0.925。 ROC curve about pre-operative and post-operative Cyfra 21-1 serum concentration. A: ROC curve about pre-operative Cyfra 21-1 serum concentration. The AUC was 0.778±0.074 (*P*=0.006). 1.66 ng/mL was defined as the cut-off value when the sensitivity was 0.889 and the specificity was 0.677; B: ROC curve about post-operative Cyfra 21-1 serum concentration. The AUC was 0.804±0.097 (*P*=0.003). 2.66 ng/mL was defined as the cut-off value when the sensitivity was 0.667 and the specificity was 0.925.

### 2.4 血清Cyfra 21-1与术后随访

无复发的93例患者中，术后血清Cyfra 21-1水平与术前相仿（*P*=0.375）。亚组分析显示，术前血清Cyfra 21-1水平较高（≥1.66 ng/mL）的患者（*n*=30），其术后该值明显下降[(3.76±4.18) *vs* (1.90±0.77), *P*=0.020]。9例患者出现复发/转移，其中1例淋巴结转移，3例胸膜复发/转移，5例远处转移。其中4例患者复发前血清Cyfra 21-1高于临床正常值（5 ng/mL）。复发患者血清Cyfra 21-1水平显著高于无复发患者[(4.86±4.13) *vs* (1.59±0.73), *P* < 0.001]。

### 2.5 术后随访血清Cyfra 21-1关于复发状态的ROC曲线

作关于术后血清Cyfra 21-1的ROC曲线，得到AUC=0.804±0.097，*P*=0.003。取Cyfra 21-1值为2.66 ng/mL时，约登指数最大。此时敏感度为0.667（6/9），特异度为0.925（86/93），PPV为0.462，NPV为0.966（图 2B）。

### 2.6 对102例术后随访患者（中位随访时间28.1个月）的复发状态进行*Kaplan-Meier*分析

结果显示，3年无复发率（recurrence free rate, FFR）在组织学类型（thymoma *vs* tca, 96.5% *vs* 71.6%, *P* < 0.001）、手术切除状态（R0 *vs* R1-R2, 94.9% *vs* 50.0%, *P*=0.001）及血清Cyfra 21-1水平（low *vs* high, 97.9% *vs* 79.0%, *P*=0.001）均有显著差异（表 2、图 3）。

**2:** 3年无复发率的*Kaplan-Meier*分析 *Kaplan-Meier* analyses of 3-year FFR

	Histotype			Resection status			Cyfra 21-1a	
	Thymoma	Tca		R0	R1+R2		High	Low
FFR (3-year)	96.5	71.6		94.9	50.0		79.0	97.9
*P*	0.000			0.000			0.001	
^a^Median Cyfra 21-1 (1.66 ng/mL) was used to group patients into high and low Cyfra 21-1 cohorts.								

**3:**
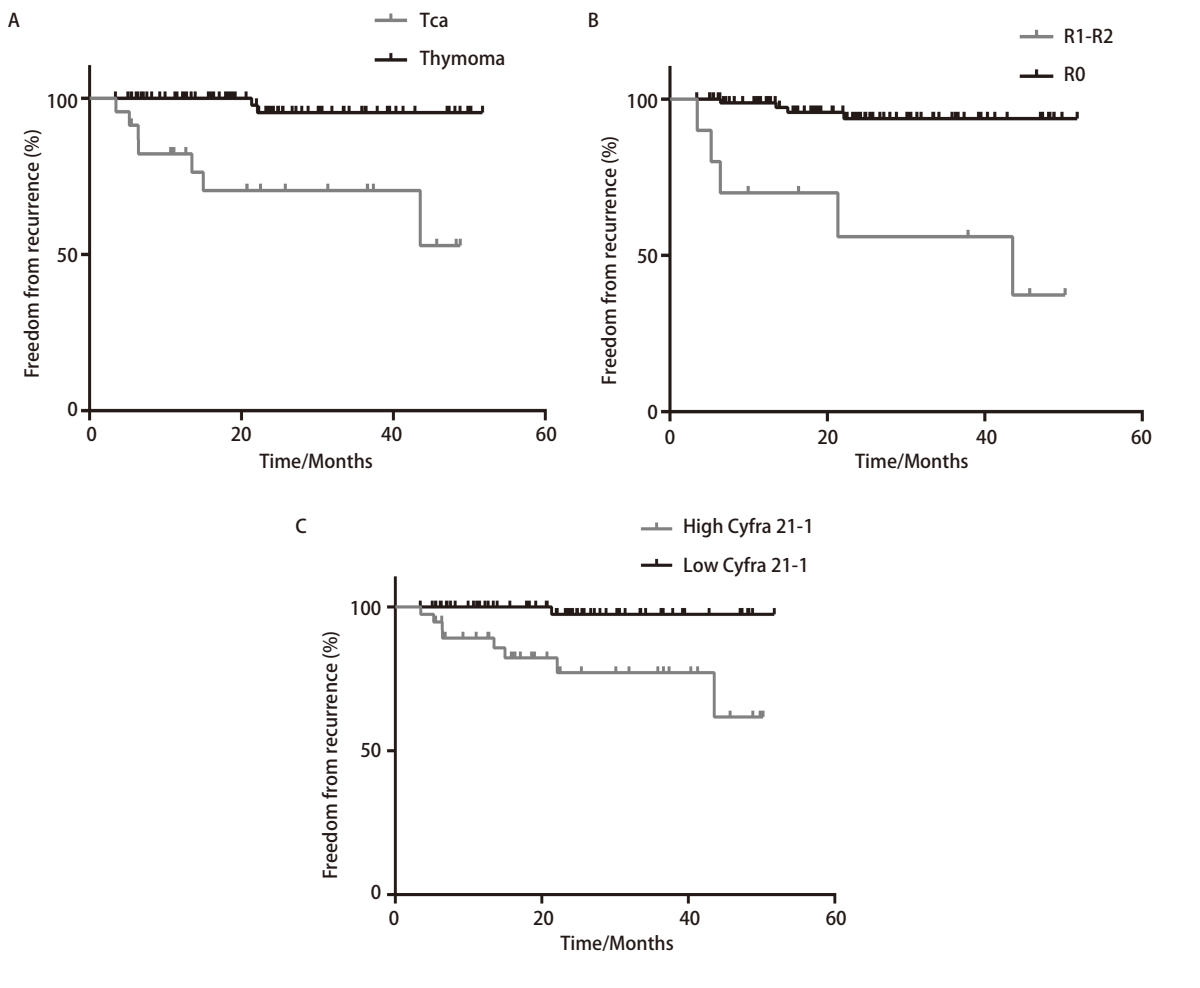
组织学类型、手术切除状态、血清Cyfra 21-1水平对于累积3年无复发率的影响。A：胸腺瘤累积3年无复发率高于胸腺癌（96.5% *vs* 71.6%, *P* < 0.001）；B：完整切除组累积3年无复发率高于非完整切除组（94.9% *vs* 50.0%, *P* < 0.001）；C：高Cyfra 21-1组累积3年无复发率低于低Cyfra 21-1组（97.9% *vs* 79.0%，*P*=0.001）。 Prognostic values of histotype, resection status and Cyfra 21-1 were assessed about FFR. A: 3-year FFR in thymoma group was significantly higher than that in thymic carcinoma group (96.5% *vs* 71.6%, *P* < 0.001); B: 3-year FFR in R0 group was significantly higher than that in R1-2 group (94.9% *vs* 50.0%, *P* < 0.001); C: 3-year FFR in high Cyfra 21-1 group was significantly lower than that in low Cyfra 21-1 group (97.9% *vs* 79.0%, *P*=0.001).

### 2.7 纳入患者年龄、性别、肿瘤长径对随访患者复发状态进行*Cox*回归分析

结果如表 3所示。在单因素*Cox*回归分析中，组织学类型（Tca）、手术切除状态（R1-R2）、术前血清Cyfra 21-1水平（high）、肿瘤T分期（T4）、存在淋巴结转移（N1-N2）均为术后复发的危险因素。经多因素分析后，仅组织学类型（Tca）和手术切除状态（R1-R2）为胸腺肿瘤患者术后复发的独立危险因素（*P*值分别为0.049及0.005）。

**3:** 单因素及多因素*Cox*回归分析 Univariable and multivariable *Cox* regression analyses

	Univariable model					Multivariable model			
	HR	*P*	95%CI			HR	*P*	95%CI	
			Lower	Upper				Lower	Upper
Age (continues)	1.032	0.310	0.971	1.097					
Gender (male *vs* female)	0.962	0.955	0.258	3.587					
Tumor size (continues)	1.152	0.300	0.881	1.507					
Histotype (thymoma *vs* tca)	0.069	0.001	0.014	0.332		0.087	0.049	0.008	0.989
Resection status (R0 *vs* R1-R2)	0.061	0.000	0.016	0.232		0.034	0.005	0.003	0.364
Cyfra 21-1^a^ (low *vs* high)	0.064	0.010	0.008	0.514		0.157	0.108	0.016	1.498
T stage (T1-T3 *vs* T4)	0.062	0.000	0.016	0.236		3.949	0.294	0.304	51.22
N stage (N0 *vs* N1-N2)	0.171	0.030	0.035	0.840		0.174	0.080	0.025	1.231
M stage (M0 *vs* M1a)	0.330	0.298	0.041	2.663					
^a^Median Cyfra 21-1 (1.66 ng/mL) was used to group patients into high and low Cyfra 21-1 cohorts.									

## 3 讨论

细胞角蛋白19是构成细胞的酸性蛋白之一，广泛分布于正常上皮细胞的胞浆中。细胞癌变时，细胞角蛋白19经由蛋白酶降解或细胞凋亡降解成片段，其中之一因能结合两种单抗（KS19.1和BM19.21）而被命名为Cyfra 21-1。在多种肿瘤中均有不同程度的Cyfra 21-1升高。在非小细胞肺癌（non-small cell lung cancer, NSCLC）患者中，Cyfra 21-1作为诊断、治疗及预后监测的价值被越来越多的研究所肯定。也有学者将其应用于原发性肝癌、胆管癌、膀胱癌、食管癌等多种肿瘤中并取得了不错的研究成果。

目前在胸腺肿瘤领域尚无特异性的肿瘤标志物，血清Cyfra 21-1仅见个案报道。本研究回顾性分析了上海市胸科医院的159例胸腺肿瘤患者，发现在胸腺癌患者中，术前血清Cyfra 21-1水平显著高于其他患者，且在T4期患者和无法R0切除的患者中，血清Cyfra 21-1水平亦表现为高于其他患者，这些都提示，在胸腺肿瘤分期的术前评估中，除影像学资料[胸部CT、胸部磁共振成像（magnetic resonance imaging, MRI）、PET/CT等]以外，血清Cyfra 21-1亦可作为参考指标之一。血清Cyfra 21-1的升高，可能提示了肿瘤分期较晚、肿瘤恶性程度较高以及无法R0切除的可能性较大。最终经由多因素*Cox*回归分析，肿瘤组织学类型及手术切除状态是患者术后复发的独立危险因素，这与国际上的报道相符。目前较为明确的胸腺肿瘤患者预后的三大独立危险因素中^[5, 6]^，肿瘤分期及组织学类型在患者就诊时已固定，因此当影像学（如胸部CT/MRI等）及血清学（如Cyfra 21-1等）检查提示肿瘤无法完整切除的可能性较大时，可考虑行术前诱导治疗，以期通过诱导治疗获得降期，提高手术完整切除的可能性，使此类患者能从中获益^[8]^。

在随访无复发的患者中，术前血清Cyfra 21-1水平较高者，术后Cyfra 21-1下降较为显著。这可能是由于肿瘤切除后，肿瘤负荷的减轻使得Cyfra 21-1的生成减少。

除此之外，本研究发现当术前血清Cyfra 21-1≥1.66 ng/mL时，其对患者随访过程中的复发状态有一定指导意义。虽然该值的阳性预测值较低（0.211），但其阴性预测值较高（0.984），可借此猜测，术前血清Cyfra 21-1水平低于该值的患者术后复发的风险相对较小。进一步对患者随访过程中的血清Cyfra 21-1进行分析，将2.66 ng/mL作为cutoff值时，提示胸腺肿瘤术后复发的敏感度为0.667，特异度为0.925，阳性预测值为0.462，阴性预测值为0.966。这提示我们，如果在随访过程中，影像学上出现新发的可疑复发病灶而难以判断时，可以借助血清Cyfra 21-1值的高低，来辅助评估复发的可能性。因此我们认为，血清Cyfra 21-1的检测在一定程度上可作为胸部CT随访的补充，尤其当影像学上出现早期难以判断的可疑病灶时，血清Cyfra 21-1的值可为临床医生提供一定的帮助。

在现行的胸腺肿瘤随访指南中，欧洲胸外科协会（European Society of Thoracic Surgeons, ESTS）胸腺工作小组推荐胸腺肿瘤患者术后3年内每3个月-6个月行胸部CT随访，术后第4年开始每年行一次胸部CT检查，终生复查。相对应地，ITMIG建议，术后5年内每年行一次胸部CT检查，随后改成每年一次的胸部CT/胸部X线交替检查，直至术后第11年，之后可以终生以胸部X线复查。另外，局部晚期（Ⅲ期、Ⅳa期）、胸腺癌、非R0切除或合并有其他高危因素的患者建议术后3年内每半年胸部CT随访^[7, 10]^。基于此，我们建议可将血清Cyfra 21-1的检测加入到术后复查中，提高复发患者的检出率，及时干预，以期改善复发患者的预后。

本研究具有一定的局限性。研究对象的选取上，经过诱导治疗后再手术的患者未予纳入，这是出于对诱导治疗可能改变肿瘤分期造成偏倚的考虑。其次，本研究为回顾性研究，难以避免地会产生选择偏倚及信息偏倚。加之单中心、罕见病的限制，本研究结果在其他中心及地区的应用效果仍需进一步验证。另外，因胸腺肿瘤相对惰性，研究周期长于其他恶性肿瘤，本研究随访时间相对较短，可能不能很好地代表其肿瘤学特性，并且在随访期间，未出现死亡病例，因此仅仅分析了FFR，而无法对OS进行相应的分析，这也是接下来的工作中需要努力的方向之一。

综上所述，治疗前患者血清Cyfra 21-1水平较高，有助于提示肿瘤分期较晚、肿瘤恶性程度较高，或可提示术后复发的风险升高。故当术前影像学（如胸部CT/MRI等）及血清学（如Cyfra 21-1等）检查提示肿瘤无法完整切除的可能性较大时，可考虑行术前诱导治疗，以期通过诱导治疗获得降期，提高手术完整切除的可能性，使此类患者能从中获益。而在术后随访过程中，除ESTS^[7]^及ITMIG^[10]^推荐的随访方案外，可以考虑加入血清Cyfra 21-1的监测，将可能有助于提高复发患者的检出率，及时干预，改善患者预后。

## References

[1] Engels EA (2010). Epidemiology of thymoma and associated malignancies. J Thorac Oncol.

[2] de Jong WK, Blaauwgeers JL, Schaapveld M (2008). Thymic epithelial tumours: A population-based study of the incidence, diagnostic procedures and therapy. Eur J Cancer.

[3] Fang W, Fu J, Shen Y (2016). Management of thymic tumors-consensus based on the chinese alliance for research in thymomas multi-institutional retrospective studies. J Thorac Dis.

[4] Falkson CB, Bezjak A, Darling G (2009). The management of thymoma: a systematic review and practice guideline. J Thorac Oncol.

[5] Rea F, Marulli G, Girardi R (2004). Long-term survival and prognostic factors in thymic epithelial tumours. Eur J Cardiothorac Surg.

[6] Kondo K, Monden Y (2003). Therapy for thymic epithelial tumors: a clinical study of 1, 320 patients from Japan. Ann Thorac Surg.

[7] Huang J, Detterbeck FC, Wang Z (2014). Standard outcome measures for thymic malignancies. J Thorac Oncol.

[8] Wei Y, Zhitao G, Shen Y (2016). Preoperative induction therapy for locally advanced thymic tumors: a retrospective analysis using the ChART database. J Thorac Dis.

[9] Kondo K, Yoshizawa K, Tsuyuguchi M (2004). WHO histologic classification is a prognostic indicator in thymoma. Ann Thorac Surg.

[10] Ruffini E, Detterbeck F, Van RD (2014). Tumours of the thymus: a cohort study of prognostic factors from the European Society of Thoracic Surgeons database. Eur J Cardiothorac Surg.

